# Supplementary material for: Measuring individual semantic networks: A simulation study
Source: PLoS One. 2025 Aug 11;20(8):e0328712. doi: 10.1371/journal.pone.0328712 (PMC12338769; doi:10.1371/journal.pone.0328712)
Supplement: S1 Table — Parameter pairs for p and r used in generating individual semantic ground-truth networks along with mean values (standard deviations) of network measures for each parameter pair over all 10 iterations. Note that all individualized networks have 13,486 words as nodes. (PDF) [file pone.0328712.s001.pdf]

Measuring individual semantic networks: A simulation study

Samuel Aeschbach[1,2*], Rui Mata[2], Dirk U. Wulff[1,2]

1 Center for Adaptive Rationality, Max Planck Institute for Human Development, Berlin, Germany
2 Center for Cognitive and Decision Sciences, University of Basel, Switzerland

* aeschbach@mpib-berlin.mpg.de

p	r	**Number of Edges**	**Average Strength**	**Average CC**	**ASPL**	**Modularity**
0	1	11,647,864.3 (8.56)	439.30 (2.7e-4)	0.31 (2.4e-6)	1.20 (1.2e-6)	0.29 (0.0014)
0	0.875	12,922,855.4 (1,052.27)	489.61 (0.04)	0.31 (1.5e-5)	1.17 (5.4e-5)	0.27 (8.7e-4)
0	0.75	14,196,583.3 (1,329.00)	539.89 (0.05)	0.31 (1.8e-5)	1.14 (6.7e-5)	0.25 (3.7e-4)
0	0.625	15,472,038.6 (1,572.36)	590.21 (0.05)	0.32 (3.0e-5)	1.12 (6.8e-5)	0.24 (0.0013)
0.045	0.5	16,380,564.1 (1,714.82)	629.84 (0.05)	0.31 (3.7e-5)	1.09 (3.1e-4)	0.22 (0.0024)
0.125	0.875	11,797,253.6 (1,273.34)	448.93 (0.04)	0.28 (1.9e-5)	1.16 (1.3e-4)	0.27 (6.8e-4)
0.125	0.75	13,107,278.8 (1,088.07)	502.60 (0.05)	0.28 (2.4e-5)	1.12 (2.3e-4)	0.25 (7.9e-4)
0.125	0.625	14,418,551.5 (1,092.19)	556.33 (0.03)	0.29 (4.2e-5)	1.09 (2.7e-4)	0.23 (0.0011)
0.175	0.5	15,320,865.0 (1,663.24)	597.07 (0.06)	0.28 (3.7e-5)	1.06 (3.1e-4)	0.21 (8.1e-4)
0.225	0.375	16,249,904.7 (1,974.19)	639.58 (0.08)	0.28 (6.0e-5)	1.03 (2.8e-4)	0.20 (0.0020)
0.25	0.8	11,477,259.1 (1,793.31)	441.38 (0.08)	0.25 (4.8e-5)	1.13 (3.7e-4)	0.25 (5.7e-4)
0.25	0.7	12,552,749.0 (2,168.98)	486.47 (0.08)	0.25 (2.8e-5)	1.10 (3.2e-4)	0.23 (9.5e-4)
0.3	0.55	13,745,838.6 (2,162.77)	539.54 (0.08)	0.24 (5.3e-5)	1.06 (3.3e-4)	0.21 (0.0018)
0.3	0.45	14,832,224.7 (1,576.35)	585.35 (0.08)	0.25 (3.7e-5)	1.04 (4.2e-4)	0.20 (0.0015)
0.375	0.3	15,860,879.6 (1,754.34)	633.34 (0.07)	0.24 (5.5e-5)	1.00 (4.1e-4)	0.18 (0.0021)
0.375	0.7	11,461,968.4 (1,925.86)	446.87 (0.06)	0.22 (3.9e-5)	1.09 (3.9e-4)	0.22 (0.0026)
0.375	0.625	12,286,395.2 (1,873.39)	481.84 (0.06)	0.22 (4.5e-5)	1.07 (4.2e-4)	0.21 (0.0022)
0.45	0.45	13,581,744.8 (2,426.45)	540.09 (0.08)	0.21 (4.2e-5)	1.03 (4.5e-4)	0.18 (0.0024)
0.5	0.325	14,556,886.7 (2,258.16)	583.04 (0.09)	0.21 (3.1e-5)	1.00 (4.2e-4)	0.16 (0.0017)
0.55	0.2	15,534,136.0 (2,700.23)	625.21 (0.10)	0.21 (3.3e-5)	0.98 (5.1e-4)	0.14 (7.6e-4)
0.5	0.625	11,197,705.3 (2,530.02)	440.74 (0.10)	0.19 (6.2e-5)	1.07 (5.9e-4)	0.20 (1.8e-4)
0.55	0.5	12,163,104.1 (2,186.83)	482.89 (0.07)	0.18 (5.6e-5)	1.04 (3.8e-4)	0.17 (0.0014)
0.625	0.325	13,487,018.7 (1,990.79)	539.07 (0.08)	0.18 (4.0e-5)	1.01 (5.8e-4)	0.14 (0.0015)
0.7	0.15	14,803,873.9 (2,174.57)	591.70 (0.07)	0.18 (2.1e-5)	0.99 (4.1e-4)	0.12 (9.2e-4)
0.75	0	15,984,546.9 (2,066.59)	636.42 (0.08)	0.18 (2.7e-5)	0.98 (5.9e-4)	0.09 (5.1e-4)

Table S1. Individual network generation parameters and network measures. Parameter pairs for p and r used in generating individual semantic ground-truth networks along with mean values (standard deviations) of network measures for each parameter pair over all 10 iterations. Note that all individualized networks have 13,486 words as nodes.
