# Supplementary figures and images for: Measuring individual semantic networks: A simulation study
Source: PLoS One. 2025 Aug 11;20(8):e0328712. doi: 10.1371/journal.pone.0328712 (PMC12338769; doi:10.1371/journal.pone.0328712)

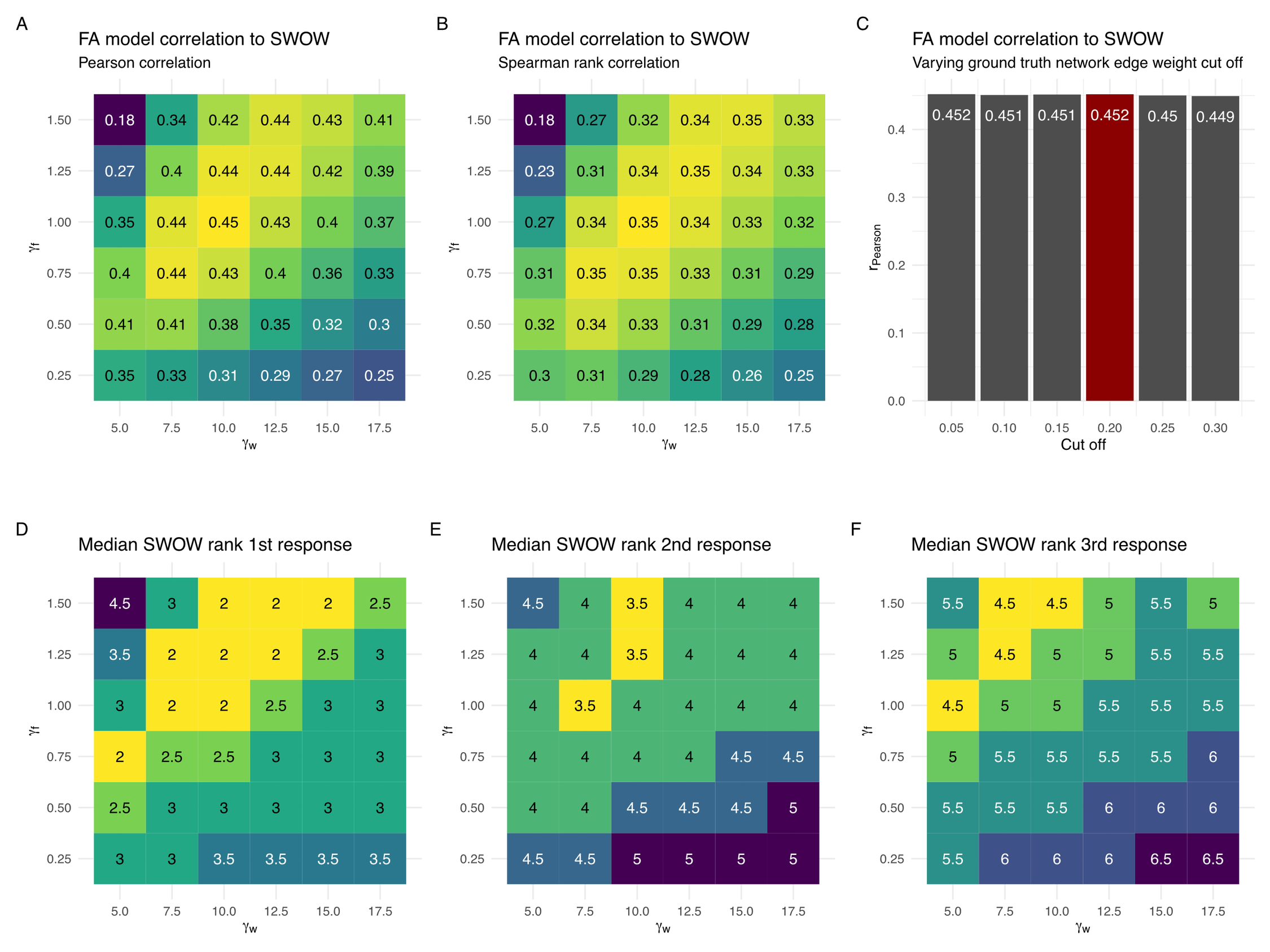

Supplement: S1 Fig — (A) Pearson correlations and (B) Spearman rank correlations of the probability distributions of first responses between model free association responses and SWOW free association norms [17]. Both correlation measures suggest γw=10 and γf=1 as best-fitting parameter values with rPearson = 0.453 and rSpearman = 0.353. (C) Pearson correlations of first responses analogously to Panels A and B, however, using fixed model parameters γw=10 and γf=1, varying semantic network ground truth minimal edge weight. This analysis shows an edge weight cut off of 0.2, as implemented in the study, to perform well in comparison to other cut offs. Free association model (D) first response, (E) second response, and (F) third response word median rank among SWOW norms [17] for the same cue word. Panels D, E, and F show the model parameters γw=10 and γf=1, as best-fitting values, to generate monotonously increasing median ranks (Med(R1) = 2, Med(R2) = 4, Med(R3) = 5). (TIF) [file pone.0328712.s002.tif]

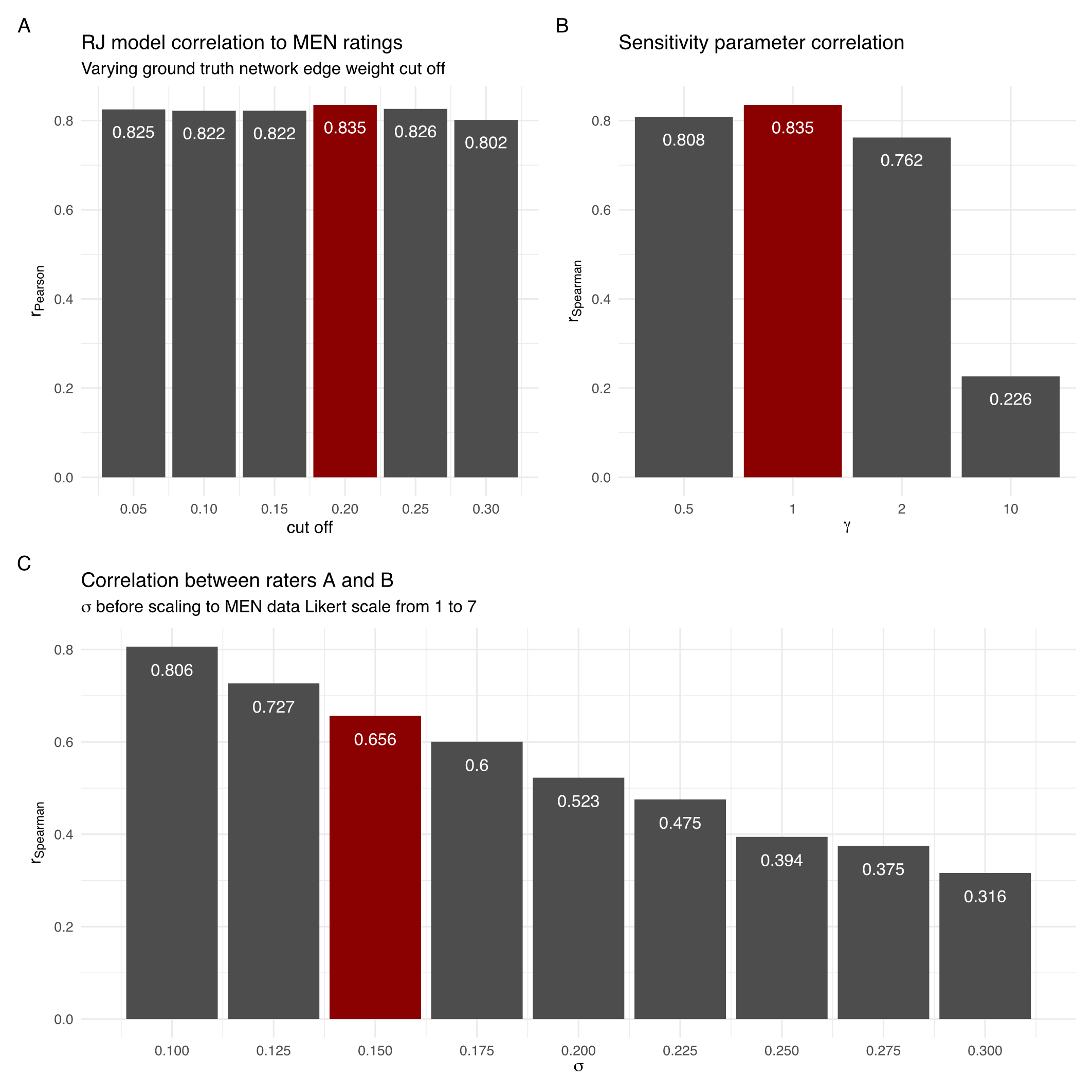

Supplement: S2 Fig — (A) Correlations between model using networks of varying lower cut offs and MEN relatedness judgment norms [35], showing 0.20 to be a good compromise between computational resource sparsity and performance. (B) Correlations between model and MEN norms [35] for varying γ values, showing γ=1 to be the best fit reproducing the behavioral ratings. (C) Tuning of model σ parameter to MEN norms [35] inter-rater reliability of rSpearman = 0.68 by simulating raters A and B and correlating their ratings, showing σ=0.15 to best approximate the reported inter-rater reliability. (TIF) [file pone.0328712.s003.tif]

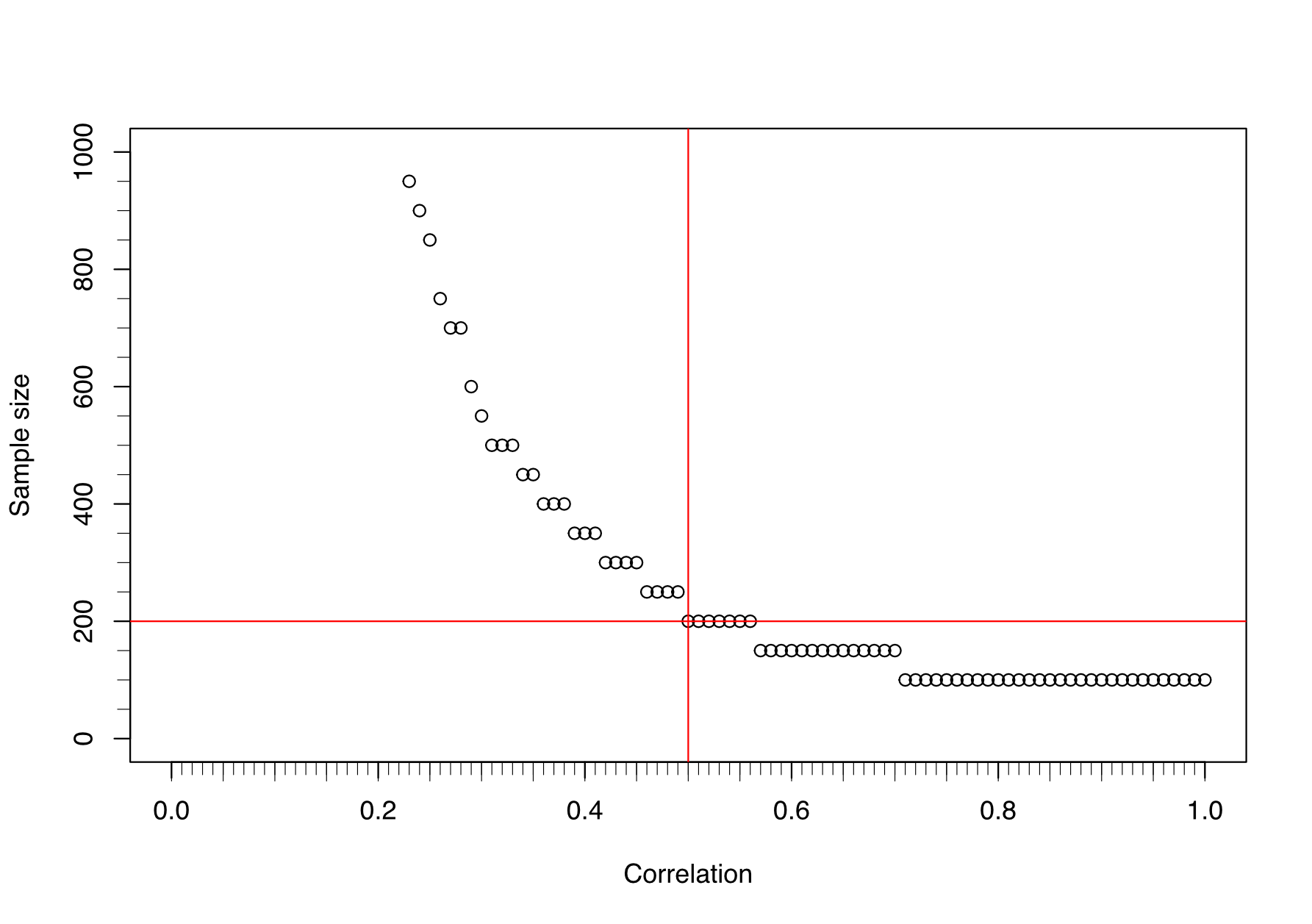

Supplement: S3 Fig — We simulated the statistical power of studies with a medium-sized true effect of Cohen’s d = 0.5 at varying levels of resolution and sample size to find a resolution of r = .5 to correspond to a power of 1−β=.8 in studies comparing samples of n = 200 using a one-sided t-test at α=.05. (TIF) [file pone.0328712.s004.tif]

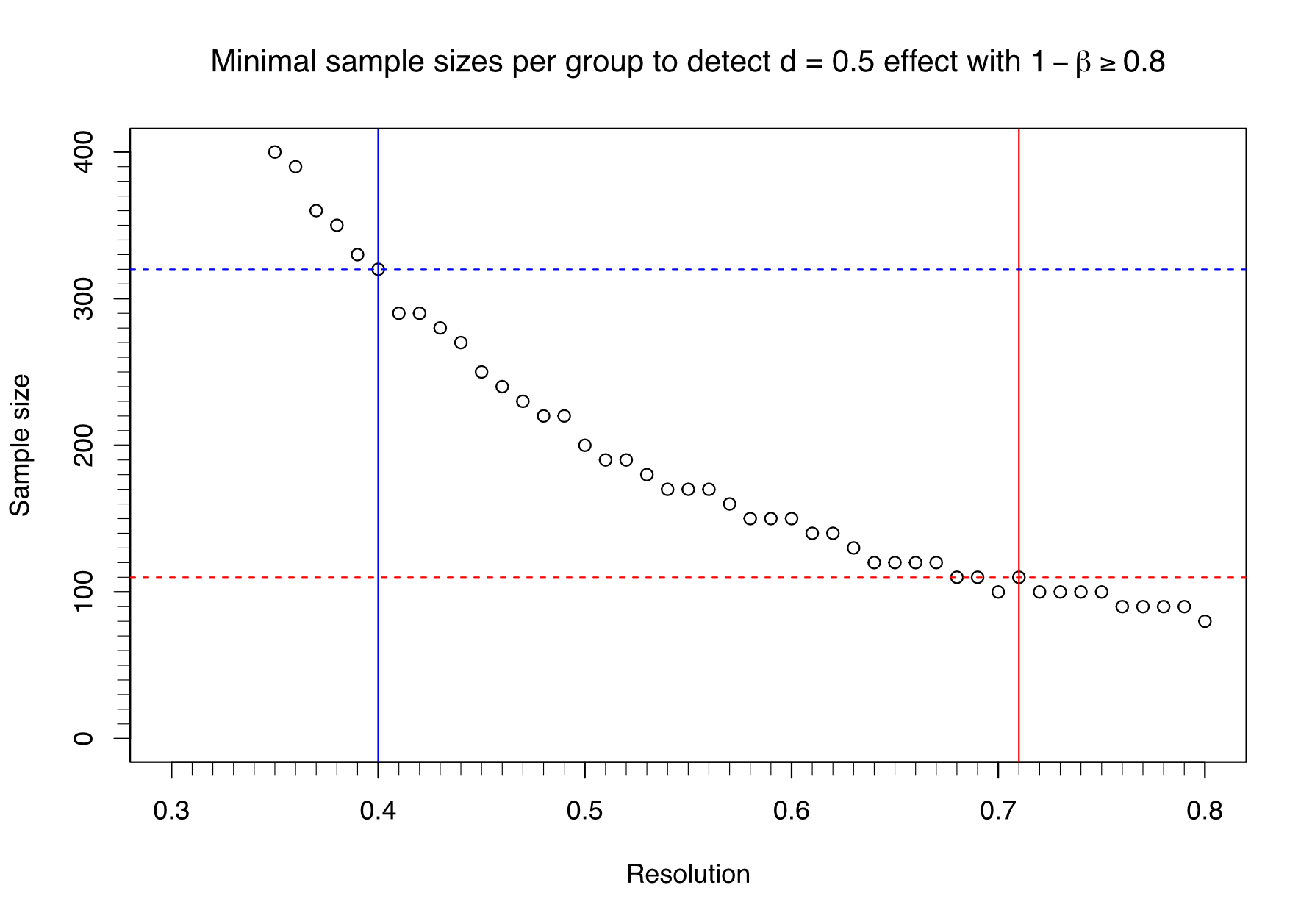

Supplement: S4 Fig — We simulated the statistical power of studies with a medium-sized true effect of Cohen’s d = 0.5 at varying levels of resolution and sample sizes to find sample sizes promoting to a power of 1−β≥.8 for study designs with resolutions r = .61 and r = .40 using a one-sided t-test at α=.05. (TIF) [file pone.0328712.s005.tif]
