# Supplementary material for: Measuring individual semantic networks: A simulation study
Source: PLoS One. 2025 Aug 11;20(8):e0328712. doi: 10.1371/journal.pone.0328712 (PMC12338769; doi:10.1371/journal.pone.0328712)
Supplement: Attachment — Submitted filename: Responses_to_reviewers.pdf [file pone.0328712.s006.pdf]

Reviewer #1: Reviewer background: statistics and linguistics

Summary: The work is a simulation study that aims to address limitations in measuring individual differences in semantic networks. They focus on constructing individual semantic networks from two behavioral paradigms: free associations and relatedness judgement tasks. Their goal is to identify certain design configurations of these semantic networks that makes comparisons between individual networks accurate and meaningful. They start by building individual ground-truth networks from a vector space model, and then they generate behavioral data from which they construct many different configurations of inferred individual semantic networks. They then compare the inferred networks with the ground truth networks to assess bias, resolution, and generalizability of network measurements. These criteria allow the researchers to identify the design configurations in which it is suitable/unsuitable to compare individual semantic networks in a meaningful way.

Overall impression: The paper is well-written and easy to understand. It is a very useful study for researchers who work with individual semantic networks, as it can contribute to more methodologically sound investigations. The results are interesting and can easily be applied. Overall it is a great paper that I think will be of great interest to a specific group of researchers.

Suggestions: Some parts of the introduction could benefit from more details. Also, some of the methodology is somewhat unclear and could benefit from more detailed explanations. I provide more specific suggestions below.

We thank Reviewer #1 for their positive evaluation of the manuscript and their considerate feedback that helped us to make improvements to the manuscript, which we believe will help readers better understand our motivation and methodology. We respond to each point raised by Reviewer #1 below.

Lines 25-26: “Recent work suggests, however, that this approach may be subject to significant limitations [9–12].” What kinds of limitations?

To clarify this, we added a pointer to the following section, where we have significantly expanded our discussion of limitations and their consequences.

"Recent work suggests, however, that this approach may be subject to significant limitations [9–12], which we discuss below."

Lines 61-71: I believe this paragraph could benefit from significant elaboration. This paragraph is essentially the main motivation for the study. I think you should offer more details about the limitations you aim to investigate. Describe the biases, how they emerge, the types of problems they can cause, and give examples. Do the same for resolution and generalizability. By elaborating on these three potential limitations, the reader is more prepared to understand the analyses that you do in the simulation study.

We expanded each of the three points considerably. We believe this section now offers a better understanding of the limitations in past research designs that motivate our simulation study.

"However, recent research also suggests that methods used in those studies suffer from important psychometric limitations. ~~First, empirical estimates likely suffer from biases due to aggregation [10] or the influence of retrieval and inference mechanisms [12].~~ First, empirical estimates of semantic networks' structural properties likely suffer from biases due to data aggregation and retrieval mechanisms [10]. Past work analyzing data from individual-level relatedness judgments found a network from aggregated responses to overestimate the average degree, clustering coefficient, and average shortest path lengths relative to individual-level networks (see analyses in supplementary material of [10]). Other work analyzing fluency data [12] has shown that inference mechanisms employing unsuitable retrieval mechanisms introduced distortions in the estimation of key network measures, highlighting how retrieval mechanisms can introduce biases, even when measuring networks at the individual level [21, 26]. These biases from aggregation and retrieval mechanisms present serious challenges to comparing results between studies. ~~Second, estimates of individual-level semantic networks likely suffer from a low resolution of true differences in estimated differences of network parameters [9], especially when considering the properties of the whole network [11].~~

Second, estimates of individual-level semantic networks likely suffer from low resolution, that is, they lack precision and are only weakly correlated with the true network parameters [9]. Attempting to predict individual differences in semantic tasks in the lab, past work has found individual-level networks to perform no better than a network from aggregate data [9]. This result was attributed to collecting a

single rather than multiple sets of responses per cue, limiting the precision of individual-level network estimates and, in turn, resolution. Another study comparing semantic networks inferred from multiple types of behavioral data from the same individuals found that network-wide measures are often unreliable, negatively affecting resolution [11]. However, resolution is critical to deriving solid conclusions about group and individual differences, which has been a core interest in the analysis of semantic networks [1, 21]. ~~Third, many results concerning the structure of semantic representations are based on highly specific contents in the mental representation, such as animals, tools, or foods (e.g., [12]). The semantic representations of such specific contents could be structured differently than the semantic representations as a whole. [9]~~
Third, many results concerning the structure of semantic representations are based on highly specific contents in the mental representation, such as animals, tools, or foods (e.g., [11, 12]), potentially limiting their generalizability. The semantic representations of any specific content are potentially structured differently from other contents or the semantic representations as a whole [9]. The highly concrete words in such specific categories (e.g., animals, countries) likely further compound this problem. Concreteness interacts with many aspects of cognitive performance [27], including word retrieval from memory [28]. Insights from semantic networks of concrete words might therefore not generalize. However, generalizability determines both the feasibility of cost-effectively constructing semantic networks and the broader applicability of findings from previous empirical semantic network research."

Section "Generating individual ground-truth networks": You describe how the networks are created, but it could be helpful to the reader to elaborate on the meaning of these ground-truth networks. What do they represent? Does each network represent a hypothetical ground-truth mental-lexicon of an individual person? (This is how I understood it). It could be helpful to provide a summary table of the statistics of the 250 networks (nodes, edges, density, clustering).

We expanded the last paragraph of the section "Generating individual ground-truth networks" to elaborate on the meaning of these ground-truth networks, making it more explicit that the 250 ground-truth networks indeed represent different individuals. We further added summary statistics of the 250 individualized ground-truth networks for each of the 25 unique parameter combinations of r and p to Table S1 and refer to it in the same paragraph.

"We generated individualized networks by running the algorithm 10 times for each of 25 combinations of p ∈ [0, 0.75] and r ∈ [0, 1] for which the networks' average strength and clustering were largely independent ~~(see S1 Table).~~ The 25 combinations have been selected such that the networks' average strength and clustering were uncorrelated (r = .009), facilitating an independent evaluation of these two measures. The resulting 250 ground-truth networks serve as a model of individuals with structurally different semantic representations. S1 Table shows a summary of network measures for each parameter value pair."

Section "Simulating behavioral data": This part is somewhat unclear to me. Where are the edge weights coming from in equations 1 and 2? Are they coming from the fully connected vector space model ground-truth network? This is what I understood. Also, here it could be helpful to discuss what this data represents. It represents essentially "sampling" from the ground-truth mental lexicons of an individual person, and that sampling is done by asking questions in a behavioral experiment. Therefore the methods you describe to generate these data (free associations and relatedness judgement) are simulations of this sampling (This is how I understand it).

The edge weights in Equations 1 and 2 are derived from the individualized ground-truth networks, which are generated using the algorithm described in "Generating individual ground-truth networks" from the fully connected network derived from the vector-space model. The individualized networks are not fully connected for two reasons: First, due to the individualization algorithm randomly moving edge weights to different edges, deleting edge weights in some cases; second, due to the final step where we remove edges with weights below 0.2. Indeed, we think of the simulation of behavioral data from the individualized networks as simulating the process of retrieving free associations or relatedness judgments from an individual's semantic representation. We added a sentence to the first paragraph of "Simulating behavioral data" to make this more explicit.

"To simulate behavioral data from the individualized semantic networks, we used cognitively plausible retrieval mechanisms of free associations and relatedness judgments tuned to publicly available data. By using these networks as the basis to simulate behavioral data from, the resulting simulated data are a product of the individually different semantic networks, analogous to behavioral data recorded from

humans that is based on each person's individually different semantic representation. This work did not involve collecting human data; no ethics approval was required."

Section "Design configurations": At the end of this section, I think it would be useful to summarize the number of total configurations you have, which you don't do until later, in the paragraph immediately before the Results. Here, a summary table could be helpful as well.

We added a short paragraph to the end of this section to summarize the total number of study design characteristics and how it is calculated. In addition, we now refer to Fig 1 Panel 2, which contains a visual representation of the study design characteristics and their levels.

"Overall, this results in design configurations varying on three levels of cue set size, three levels of cue set type, three numbers of responses per cue collected, and two behavioral data types, amounting to 54 design configurations (see also, Fig 1 Panel 2)."

Section "Evaluating network recovery": This part is unclear to me. How are the comparisons between inferred networks and ground-truth networks made? Is one inferred network compared to one ground-truth network? What exactly are M and M-hat? What measures are they? Are they calculated on individual networks, one inferred network of a given configuration and one ground truth sub-network reduced to match that configuration?

Networks are compared for each simulated subject individually: each simulated subject's inferred network is compared to the same simulated subject's ground-truth network. M and M-hat are stand-ins for any of the network measures described in the section "Network measures." As suggested, the measures (e.g., clustering coefficient) are calculated separately for each individual's inferred and ground-truth networks, with the latter being reduced to the relevant sub-network. This yields 2,500 inferred and ground-truth values per measure and design configuration, entering the calculation of the three criteria. We modified the section "Evaluating network recovery" to more explicitly state on which level the comparisons are made and what M and M-hat denote.

"Our evaluation of network recovery relies on three criteria: bias, resolution, and generalizability. We evaluate each criterion by comparing, for each individual i and

each network measure M, the measure of their inferred network ($\hat{M}_i$), with that of their ground-truth network (M_i). We evaluate bias as the geometric mean of the ratio between the inferred and ground-truth network measures minus one, i.e.,

$$Bias = \left(\prod_{i=1}^{n} \frac{\hat{M}_i}{M_i}\right)^{\frac{1}{n}} - 1."$$

Nice paper!!

Thank you!

Reviewer #2: Summary

- This research investigates the methodological challenges in measuring individual semantic networks through behavioural paradigms (of free associations and relatedness judgments). Using comprehensive recovery simulations, the authors evaluate how different study design parameters affect the bias, resolution and generalisability of semantic network measurements. The study certainly makes a significant contribution by systematically assessing the conditions under which semantic networks can be reliably inferred from behavioural data.

Strengths

- The methodology used is rigorous, examining multiple design parameters, with any shortcomings that become apparent reading through being clearly addressed in the limitations and future work. There are also practical implications for future empirical work, making this a useful reference work in the field.
- The evaluation of network measures is comprehensive, although given the part focus on broad, narrow and mixed cue sets, it is regrettable as a reader that remaining matters such as network diameter could not be included in this work.
- The insights on the generalisability of these network measures is a particularly impactful consideration, especially to identify the limitations of previous works.

We thank Reviewer #2 for the positive evaluation of our manuscript and the considerate suggestions that helped us make improvements to the manuscript, which we believe made it stronger.

Issues

- Page 4, Section “Generating individualized ground-truth networks” – There should be a simple justification/validation for the choice of parameter range of `p` and `r` to make obvious there are no complex underlying reasons.

We have extended the last paragraph of the section “Generating individualized ground-truth networks” to provide a clearer explanation of why we chose the specific ranges and values of “p” and “r”. The parameter ranges were selected to maximize the variance of average strength and clustering of the individualized ground-truth networks, while keeping the two uncorrelated. We added summary statistics of network measures for the ground-truth networks to S1 Table.

> “We generated individualized networks by running the algorithm 10 times for each of 25 combinations of p ∈ [0, 0.75] and r ∈ [0, 1] ~~for which the networks' average strength and clustering were largely independent (see S1 Table)~~. The 25 combinations have been selected such that the networks' average strength and clustering were uncorrelated (*r = .009*), facilitating an independent evaluation of these two measures. The resulting 250 ground-truth networks serve as a model of individuals with structurally different semantic representations. S1 Table shows a summary of network measures for each parameter value pair.”

- Page 5, Given that it is the basis of the synthesised responses and further experiments, the cognitive plausibility of the retrieval mechanisms used in the simulations could benefit from stronger theoretical grounding, or perhaps elaboration of the references to justify it. However, this is considered in the discussion, especially mentioning the potentially disparate nature of individual semantic retrieval processes. It could be deemed a necessary simplicity for, as written controllability, when considering that including methodology from Human Modelling/Computational Rationality fields would make this work overly complex.

We agree with the reviewer that the cognitive plausibility of retrieval mechanisms is an important consideration. However, for this particular study, which centers on how study design influences network measure recovery, a detailed exploration of these mechanisms would introduce additional complexity and go beyond the scope of our contribution. To ground our simulations, we drew on literature in semantic memory search that shows how a combination of general word retrieval probability (word frequency) and specific retrieval probability based on

prior activations (relatedness in a language model) predicts the retrieval of words from memory (Hills et al., 2015; Wulff et al., 2020). Further, relatedness judgments are well predicted by semantic similarity in a language model (e. g., Bruni et al., 2014), while human judgments are inherently variable. These dimensions are included in our simulation approaches. We opted for these simpler, established simulation approaches to avoid making unfounded assumptions. We believe our conclusions regarding study design effects remain robust for two reasons: first, they are based on simulations with constant, albeit simplified, data generation processes; second, the data generation processes are tuned to empirical data to match human retrieval patterns. Our text now provides additional detail to emphasize the theoretical basis behind the data generation processes.

> "Free associations are generated by a retrieval process based on semantic similarity given by the edge weights and word frequency. Past work has shown ~~how a process combining semantic similarity and frequency accounts well for human memory retrieval [16, 33].~~ that local (specific to prior word activation) and global (non-specific to prior word activation) word properties are important to predict the retrieval of words from memory [32]. Specifically, the combination of semantic similarity (local) and word frequency (global) has been found to account well for human memory retrieval [16, 32, 33]."

> "Relatedness judgments are generated based on truncated normal distributions around the corresponding nodes' edge weights. ~~We scaled and restricted the judgments to the interval [1, 20] to generate responses between 1 and 20 in line with Wulff et al. (2022) [10].~~ This assumes a minimal process in which relatedness judgments directly reflect semantic similarity within a judge's mental representation, translated into somewhat noisy ratings on a scale from 1 to 20 (following Wulff et al., 2022 [10])."

- Page 9, Figure 3. Resolution caption has an error (assumed typographical) in the range for teal tiles "0 ≥ r < .5" -> "0 <= r < .5".

This was indeed a typographical error, which we now corrected. Thank you for spotting it.

> "Resolution of the recovery is defined as good for values r ≥ .5 (yellow tiles), positive for values ~~0 ≥ r < .5~~ 0 ≤ r < .5 (teal tiles), and negative r < 0 (purple tiles)."

Nitpicks (These do not need to be addressed)

- The discussion could address potential limitations of using fastText embeddings as ground truth.

The discussion now includes an extended point on limitations of our simulation's ground truth assumptions, and references a recent study investigating differences between word embedding models, including fastText.

> "First, our general and individualized ground-truth networks are based on assumptions that may be inconsistent with the true representations in people's minds. Our understanding of the structure and content of mental representations is still limited [21]. Moreover, recent work demonstrates that semantic representations derived from different sources—such as text, behavioral, or brain data—systematically diverge in the information they encode, with behavior-based embeddings in particular capturing unique aspects of psychological meaning compared to the text-based ground truth used in our simulation [43]. Together with the challenges highlighted by this investigation, it is currently likely that there are other equally or more justified sets of assumptions. Future research should, therefore, evaluate the recovery of network measures under different ground-truth assumptions, in particular, by using different base representations and algorithms to individualize networks."

- There was one sentence thought a little vague: "the networks' average strength and clustering were `largely` independent" perhaps could be more concretely communicated, but it's easily understood so no need to change.

In addition to revising this paragraph and sentence, we added the correlation between the two variables in parentheses to quantify the dependence.

> "We generated individualized networks by running the algorithm 10 times for each of 25 combinations of $p \in [0, 0.75]$ and $r \in [0, 1]$ ~~for which the networks' average strength and clustering were largely independent (see S1 Table)~~. The resulting 250 ground-truth networks serve as a model of individuals with structurally different semantic representations. The 25 combinations have been selected such that the

networks' average strength and clustering were uncorrelated ($r = .009$), facilitating an independent evaluation of these two measures. S1 Table shows a summary of network measures for each parameter value pair."

PLOS ONE 7 Criteria
- Original Research. ✓
- Results not published elsewhere. ✓
- The simulation methodology is rigorous and well-documented. Statistical analyses are appropriate. ✓
- Conclusions supported by data. ✓
- The writing is clear and professional throughout. ✓
- No ethical concerns as this is a simulation study. ✓
- GitHub repository with code and data links for reproduction. ✓

An exceptionally well written paper. The suggested revisions are only to strengthen an already solid piece of work.

Overall recommendation: Accept with Minor Revisions

Thank you for the praise!
